# Effect of dopant-induced local vibration modes on pressure-driven structural phase transition in Mn- and Co-doped ZnO

**DOI:** 10.1016/j.isci.2025.112560

**Published:** 2025-05-02

**Authors:** Chih-Ming Lin, Yi-Jia Tsai, Yi-Sheng Huang, Chia-Hung Hsu, Bo-Shiuan Chen, Ming-Fong Tai, Sheng-Rui Jian, Jenh-Yih Juang

**Affiliations:** 1 Department of Physics, National Tsing Hua University, Hsinchu 300, Taiwan; 2 Department of Materials Science and Engineering, I-Shou University, Kaohsiung 84001, Taiwan; 3 Department of Fragrance and Cosmetic Science, College of Pharmacy, Kaohsiung Medical University, 100 Shi-Chuan 1st Road, Kaohsiung 80708, Taiwan; 4 Department of Electrophysics, National Yang Ming Chiao Tung University, Hsinchu 300, Taiwan

**Keywords:** Chemistry, Physics, Materials science, Materials property

## Abstract

*In-situ* laser Raman spectroscopy was performed on pristine ZnO, Zn_0.98_Mn_0.02_O, and Zn_0.98_Co_0.02_O at pressures up to 18.6, 18.4, and 13.1 GPa, respectively, to explore how minute amount of Mn- and Co-doping affects the high-pressure wurtzite-to-rocksalt transition in ZnO. Pristine ZnO exhibited characteristic wurtzite phonon modes, whereas Mn- and Co-doping introduced distinct local vibrational modes that shifted differently under compression. These shifts revealed that Zn_0.98_Mn_0.02_O undergoes a unique phase transition path compared to pristine and Co-doped ZnO. By connecting local vibrational features to structural evolution at high pressures, this study demonstrates the capability of Raman spectroscopy to detect doping-induced changes in lattice dynamics. These insights advance our understanding of how local lattice effects influence phase transitions and guide the design of ZnO-based materials with tunable properties for optoelectronic and high-pressure applications.

## Highlights

• Dopant-induced local Raman modes dictate the wurtzite-to-rocksalt transition in ZnO
• Mn-doping introduces dual local modes, altering ZnO’s high-pressure transformation path
• Co-doping spawns a single local mode, slightly lowering the transition onset pressure
• Strain analysis confirms distinct pressure-driven paths for Mn- vs. Co-doped ZnO

## Graphical abstract

**Figure:**
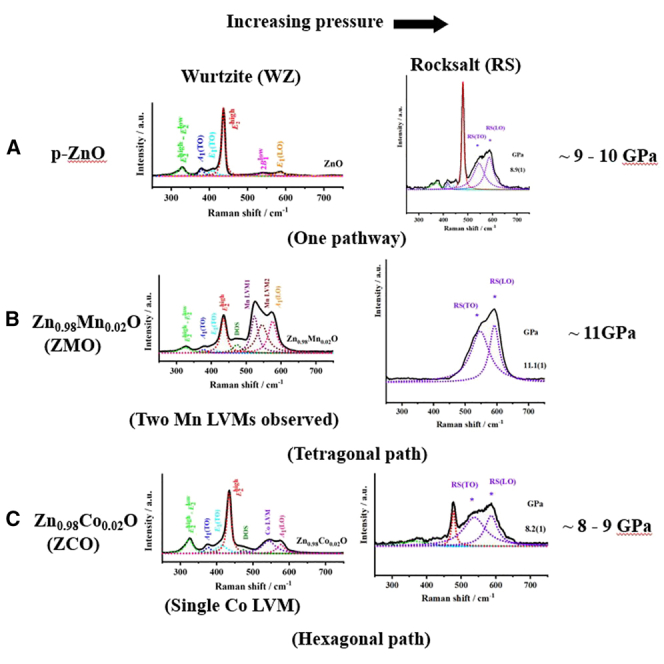

## Introduction

Among a plethora of technologically important functional materials, specifically the group IV, III-V, and II-VI binary compound semiconductors, ZnO is characterized by the wide bandgap (3.37 eV) and large exciton binding energy (60 meV), making it extremely promising for a wide range of applications including electronic and opto-electronic devices, catalysts, chemical sensors, piezoelectric transducers, and even for the transparent conducting layers for solar cells and displays. Pristine ZnO (p-ZnO) has the hexagonal wurtzite (B4) structure at ambient conditions and had been reported to undergo a phase transition to the rocksalt (B1) structure under externally applied hydrostatic pressure of approximately 9 GPa early in 1962.^1^ Since then, extensive theoretical and experimental studies have been devoted worldwide to give a rather comprehensive understanding on pressure-driven phase transitions in p-ZnO. Recently, motivated by the pursuits of further application potential in spintronics and UV-range optoelectronics, the incorporation of various extrinsic elements, especially the magnetic ones selected from the transition metals, into the wurtzite-structured (WZ) ZnO has received extensive research interests for obtaining controllable manipulations over its electronic, magnetic, and/or optical properties. The accompanying side effects on the pressure-driven structural phase transition brought about by doping, nevertheless, are equally fascinating from the fundamental point of view.

According to Saitta and Decremps,^2^ there are two possible scenarios for the path taken by pressure-driven WZ-to-rocksalt (WZ-to-RS) phase transition in ZnO: (1) the “hexagonal path”, featuring a continuous decrease in the *c/a* ratio (from *c/a* ≈ 1.6 to *c/a* ≈ 1.2) and an accompanying increase in the internal structure parameter *u* (from *u* = 0.375 to *u* = 0.5) before transforming from the WZ phase to the RS structure at the phase transition pressure (*P*_tr_); (2) the “tetragonal path”, characterized with a different distortion route by first opening up the WZ hexagonal angle accompanied by a horizontal movement of atoms to the center of the pyramid square, while the *c/a* and *u* remaining close to the typical values of ∼1.6 and ∼0.38, respectively. Based on the aforeementioned scenarios, a recent study using *in situ* angle-dispersive X-ray diffraction (ADXRD) method indicated that Zn_0.98_Mn_0.02_O (ZMO) may have taken the “hexagonal path”, while p-ZnO, Zn_0.98_V_0.02_O (ZVO), and Zn_0.98_Co_0.02_O (ZCO) followed the alternative “tetragonal path”.^3^ Although the assertions were evidently supported by the pressure dependence of the *c/a* ratio and internal structural parameters obtained by ADXRD,^3^ the underlying mechanism remains largely unexplored. In particular, why ZMO behaved so differently?

Experimentally, methods such as inelastic X-ray scattering (IXS) and inelastic neutron scattering (INS) have been commonly used in studying the pressure-dependent structural evolutions in a wide variety of materials.^4^^,^^5^^,^^6^ However, the IXS has been suffering from the relatively low energy resolution of around 10 cm^−1^ and is accessible only in large synchrotron radiation facilities.^5^^,^^7^^,^^8^^,^^9^ Likewise, INS also has an energy resolution of only ∼5 cm^−1^,^10^ in addition to large sample required, making it cumbersome to fulfill measurements under pressure.^6^ In contrast, the laser Raman spectroscopy (LRS) not only has a much higher energy resolution (≤1 cm^−1^) but also is relatively easier to access. Consequently, in recent years, combining the usage of diamond anvil cell (DAC) with the LRS has become a powerful alternative for studying pressure-dependent phonon dynamics in various semiconductors,^9^ especially on the dopant-induced local lattice distortion and electronic band structure changes,^11^ which might play an important role in the subsequent pressure-induced phase transition in ZnO.^12^ In particular, at the initial stage of phase transition the primary phonon modes and elastic constants involved are very much path dependent. Namely, the primary feature of hexagonal distortion is the compression along the *c*-axis, resulting in vertical displacement of Zn-O pairs, which is mainly reflected in the RS optical phonons. In contrast, the tetragonal distortion is more relevant to the *c*_66_ elastic constant and E_2_ optical phonons. Thus, it is expected that such subtle differences might result in substantial deviations in certain vibration modes, which might be resolvable by tracking how the respective phonon modes evolve with pressure up to the transition pressure. In this study, aiming at delineating the pivotal role played by local structural distortion on curating the path of WZ-to-RS transition under hydrostatic pressure, comparative investigations on the pressure-driven phase transition in p-ZnO, ZMO, and ZCO were conducted by combining the usage of DAC and LRS at room temperature.

In addition, we acknowledge that Mn- and Co-doped ZnO systems have indeed been extensively explored, including under ambient and moderately elevated pressures.^13^^,^^14^^,^^15^^,^^16^^,^^17^^,^^18^ Nevertheless, we emphasize that the present work provides additional insights into how these dopants influence phase transitions and defect chemistry under systematically applied high pressures. Specifically, we conducted *in situ* synchrotron XRD and Raman spectroscopy to track the structural evolution of ZnO doped with Mn and Co, thereby uncovering previously unreported details on dopant-specific effects and local structural distortions. In contrast to prior studies that Mn and Co doping were often investigated separately and under different experimental setup conditions, we analyzed both dopant species under the same high-pressure protocols. This direct comparative approach illuminates how different 3*d* transition metals uniquely affect phase transition pressures and structural pathways, offering a clearer perspective on their respective roles in modulating the host lattice. Furthermore, while earlier reports typically focused on a single dopant concentration, we explored Zn_0.98_Mn_0.02_O and Zn_0.98_Co_0.02_O to reveal how varying doping levels control high-pressure stability. By mapping these trends across multiple compositions, we demonstrate how defect accumulation and local bonding changes drive the high-pressure behavior of ZnO-based materials. Our advanced *in situ* characterization, namely by combining high-resolution synchrotron measurements with Raman spectroscopy under carefully controlled hydrostatic conditions, enables the detection of subtle dopant-induced phase transformation mechanisms that might remain inaccessible to lower-resolution or *ex situ* techniques. Finally, the implications of our findings extend to functional properties and spintronic applications. Although Mn and Co doping of ZnO have been recognized for their potential in dilute magnetic semiconductors (DMS), the effects of high pressure on magnetic ordering and electronic structure are still not fully understood.^16^^,^^17^^,^^18^^,^^19^ By showing how hydrostatic compression can modify both structural and electronic features, our results offer new perspectives on defect engineering strategies for future device applications and spintronic designs. We believe that this systematic investigation of the high-pressure phase evolution and defect chemistry in Mn- and Co-doped ZnO, carried out with advanced *in situ* approaches, adds meaningful value to the literature and may inspire subsequent studies on high-pressure phenomena, device optimization, and novel functionalities.

## Results and discussion

### Peak assignments of Raman spectra at ambient pressure

**Figure 1:**
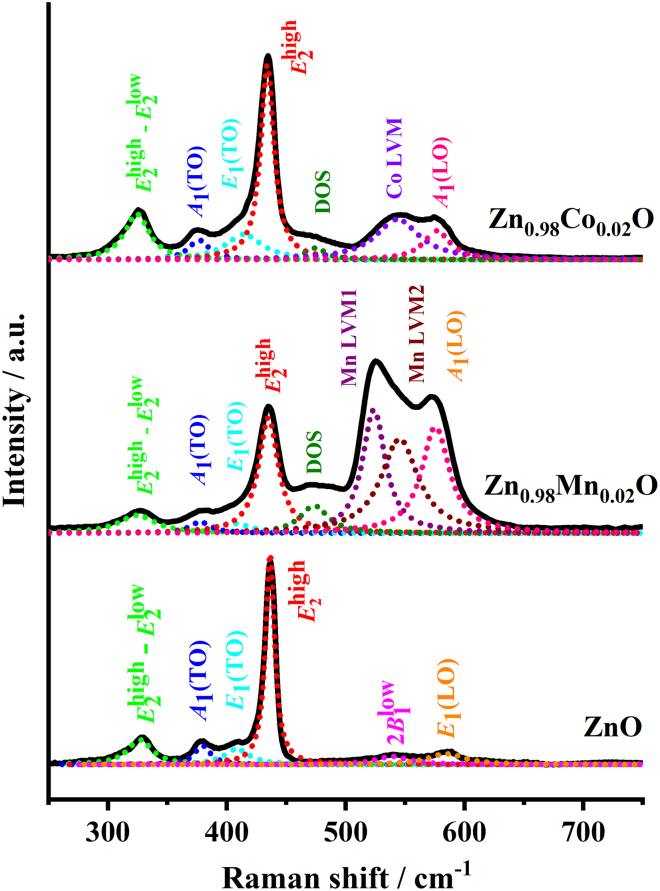
Raman data under ambient conditions Representative LRS patterns for WZ ZnO, Zn_0.98_Mn_0.02_O, and Zn_0.98_Co_0.02_O, respectively, at ambient pressure.

Figure 1 shows the Raman spectra obtained at ambient temperature and pressure together with deconvolution fittings (dotted lines) for p-ZnO, ZMO, and ZCO, respectively. The raw data obtained at ambient and a series of applied pressures in DAC are reported in the supplemental information and displayed in Figures S1A–S1C, respectively. Several interesting features are immediately evident from Figure 1. Firstly, the intensity of the principal peaks associated with the host lattice appears to be slightly different for the three samples, presumably due to the non-oriented powder samples used. Secondly, apparent extra features associated with doped impurities are clearly observed, which is also sensitive to the doping species. Theoretically, the group theory predicts that the WZ-structured ZnO, being belong to the *P*6_3_*mc* space group, could exist the following optical modes: Γ_opt_ = *A*_1_ + *E*_1_ + 2*E*_2_ + 2*B*_1_ at the Γ-point of the Brillouin zone. The *B*_1_ modes are silent modes. In contrast, the *A*_1_ and *E*_1_ modes are polar and both are Raman and infrared active, whereas the *E*_2_ modes are nonpolar and Raman active only.^12^ Both *E*_1_ and *E*_2_ phonons are associated with in-plane atomic motions on the *ab*-plane, while the atomic motions of *A*_1_ and *B*_1_ phonons occur along the *c*-axis. The WZ-structured ZnO thus has two additional Raman-active phonon doublets of Γ symmetry (*E*_2_^low^ and *E*_2_^high^), which correspond to folded (L-point) acoustic and optical phonons of zinc blende.^9^ Consequently, the Raman peaks shown in Figure 1 for p-ZnO can be tentatively assigned as followings. The peaks centering around 380, 408, and 436 cm^−1^ are the first order host lattice modes, namely *A*_1_(TO), *E*_1_(TO), and *E*_2_^high^, respectively. The band near 327 cm^−1^ is ubiquitously interpreted as a double phonon corresponding to the subtractive combination of the *E*_2_^high^ (436 cm^−1^) and the *E*_2_^low^ mode (∼100 cm^−1^) and is denoted as *E*_2_^high^-*E*_2_^low^ mode.^2^^,^^11^^,^^12^ Here, TO and LO denote transverse optical and longitudinal optical, respectively. It should be noted that the numerical values of frequencies given previously are the typical values, which can vary slightly in that the frequencies of various extraordinary phonons may change with the orientation in a uniaxial material. In addition, a mode at ∼585 cm^−1^ can be identified, whose intensity, nevertheless, is much higher than that of the *A*_1_(LO) reported by Manjon et al.^20^ obtained under ambient conditions. Moreover, the frequency observed here is also slightly higher than that identified previously in the study by Manjon et al.^20^ Alternatively, although the *E*_1_(LO) mode is forbidden in the backscattering geometry, it has been, nevertheless, observed in WZ ZnO in the forbidden *X*(*ZZ*)-*X* geometry.^21^ We thus assign the mode appearing at 585 cm^‴+^ to the *E*_1_(LO) mode. Lastly, the presence of the silent 2*B*_1_^low^ mode at ∼539 cm^−1^ has, in fact, also been predicted in previous theoretical calculations^20^^,^^21^^,^^22^ by comparing with flat regions in the dispersion curves and was attributed to some second-order structures. It is emphasized that the assignment of the observed Raman peaks for p-ZnO described previously should be considered as tentative since in the present case the incidence direction with respect to the crystal axes is not defined, and mixed modes may be obtained.^12^ Namely, unlike in single crystalline samples or in highly *c*-axis oriented films grown on specific substrates, the crystal axes of powder are randomly tilted relative to the laser excitation polarization, which, in turn, might lead to our observation of all Raman-active phonon modes. All modes listed previously may contribute to the total intensity of the broad band observed experimentally, with the mode intensity of 2*B*_1_^low^ and *E*_1_(LO) modes being weaker than others.

The doping of manganese or cobalt in ZnO is expected to alter the local crystal symmetry and, consequently, the behaviors of phonon mode. For ZMO, the band near 325 cm^−1^ could be similarly interpreted as the subtractive combination of the *E*_2_^high^ mode at 435 cm^−1^ and the *E*_2_^low^ mode, i.e., the *E*_2_^high^-*E*_2_^low^ mode. Likewise, the peaks at 378 cm^−1^, 405 cm^−1^, and 435 cm^−1^ are corresponding to the first order host lattice modes of *A*_1_(TO), *E*_1_(TO), and *E*_2_^high^, respectively. We note that the frequencies of the *E*_2_^high^-*E*_2_^low^, *A*_1_(TO), *E*_1_(TO), and *E*_2_^high^ modes for ZMO here are slightly lower than that for p-ZnO in Figure 1 and Zn_1-*x*_Mn_*x*_O (where *x* ≤ 0.03).^23^ The broad band approximately centered around 474 cm^−1^ has been attributed to the phonon density of states (DOS) associated with a disorder activated phonon mode.^24^^,^^25^^,^^26^^,^^27^ This feature has been observed in Mn implanted ZnO,^28^ which showed that the intensity of the DOS increased with the implanted Mn concentration, indicating that the broad peak is intimately correlating with the crystal disorder. Perhaps even more remarkable observation is a broad but high-intensity band within the spectra region of 500–650 cm^−1^. The Lorentzian peak fittings of this broad band indicate that it can be deconvoluted into at least three peaks centering at 524, 545, and 576 cm^−1^, respectively. Unlike the peak of ∼585 cm^−1^ aforementioned for p-ZnO, the peak at ∼576 cm^−1^ peak coincides with the frequency of the *A*_1_(LO) lattice mode,^21^ thus can be unambiguously assigned. On the other hand, the peaks at 524 and 545 cm^−1^ are obviously related to the manganese impurity. Previously, these peaks had been observed in Mn-doped ZnO and were attributed to the local vibration mode (LVM) of Mn impurity or Mn-related complexes in ZnO.^23^^,^^24^^,^^25^^,^^27^ Here, we assign the peaks near 524 cm^−1^ and 545 cm^−1^ as Mn LVM1 and Mn LVM2 modes, respectively. Considering the fact that the ionic radius of Mn^2+^ (80 pm) is larger than that of Zn^2+^ (74 pm),^28^^,^^29^ it is plausible to expect that, when Mn^2+^ occupies the Zn sites, intrinsic host-lattice defects or lattice distortions might be introduced and locally reduce the crystal symmetry. As a result of symmetry lowering, additional vibration modes, such as LVMs, and/or forbidden silent modes may appear in the obtained Raman spectra. Moreover, it is noted that the possibility that the Raman spectra at ambient conditions is resulted from the MnO clusters can be ruled out in that the crystal structure of MnO is of the NaCl type and its LO mode at 552 cm^−1^ was known to be Raman inactive.^30^ In addition, Chou et al.^31^ reported that a single, two-magnon mode for possible small MnO clusters (if any) should be observed at frequency near 503 cm^−1^, which is not seen here. Finally, we note that, since these modes seem to follow the same symmetry rule as the *A*_1_(TO) (380 cm^−1^) phonon of the p-ZnO, it is tempting to attribute those two peaks, Mn LVM1 and Mn LVM2, to the vibrations of substitutional Mn impurity rather than to the Mn-related complexes suggested previously.^23^^,^^24^^,^^25^^,^^27^

For ZCO, except for the impurity-related LVM (see in the following test), the peak assignments of the obtained Raman spectra are similar to that of ZMO sample. Namely, the band near 325 cm^−1^ is corresponding to *E*_2_^high^-*E*_2_^low^ mode, whereas the peaks at 378 cm^−1^, 417 cm^−1^, 434 cm^−1^, and the broad band approximately centering at 472 cm^−1^are designated to the host lattice modes of *A*_1_(TO), *E*_1_(TO), *E*_2_^high^, and the disorder-activated DOS mode, respectively.^11^^,^^24^^,^^32^^,^^33^ In contrast to that observed for ZMO, the broad band appearing within the frequency range of 500–600 cm^−1^ can be unambiguously deconvoluted into two peaks centering at ∼535 cm^−1^ and ∼559 cm^−1^, respectively. Similar to that observed at ∼576 cm^−1^ for ZMO, the peak at ∼559 cm^−1^ can be assigned to *A*_1_(LO) lattice mode. The frequency of the *A*_1_(LO) mode in ZCO is slightly lower than that in ZMO, which might be due to the fact that Co is heavier than Mn and/or that Co-doping might result in slight bond weakening. On the other hand, the peak at ∼535 cm^−1^ must be the LVM associating with Co impurity. However, unlike in ZMO, only one LVM is identified. Previous studies had observed similar LVM mode appearing around 539 cm^−1^,^24^ 556.9 cm^−1^,^34^ and 575 cm^−1^,^26^ respectively, and attributed it to cobalt-related complex involving, for example, another Co^2+^, an oxygen, a zinc interstitial, or even a hydrogen ion etc.^34^ Such impurity-associated complexes not only would lead to local lattice deformation but also give rise to distinct magnetic behaviors unique to the specific dopant species.^35^ The absence Raman characteristic peaks associated with the CoO (143 and 296 cm^−1^)^36^^,^^37^^,^^38^^,^^39^ and metallic Co clusters (134 cm^−1^)^30^ in Figure 1 indicates that this Co LVM may arise from the substitutional Co impurity. Moreover, considering that the ionic radius of Co^2+^ (74.5 pm) is only marginally larger than that of Zn^2+^ (74 pm),^29^ it is reasonable to speculate that the host lattice only encounters a relatively smaller distortion by the substitutional occupation of Co on Zn sites as compared to that caused by Mn doping. As a result, only one LVM is observed, which might be of relevant importance in subsequent pressure-induced WZ-to-RS phase transition. (See the following text*.*).

### Evolution of Raman spectra with increasing pressure

**Figure 2:**
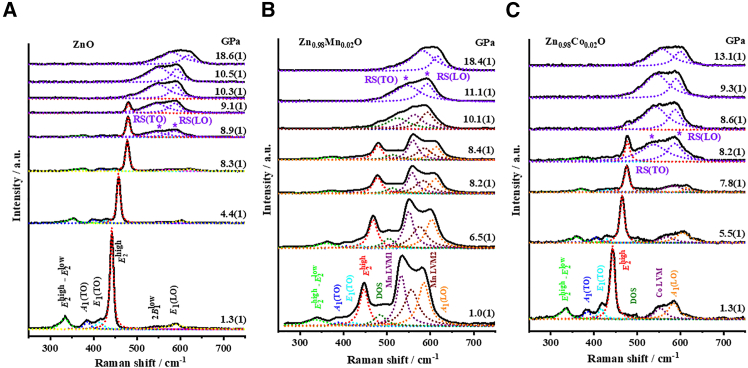
Raman data under pressure Representative LRS patterns of (A). WZ ZnO, (B). Zn_0.98_Mn_0.02_O, and (C). Zn_0.98_Co_0.02_O at elevated pressures. See also Figure S1A and Table S1, Figure S1B and Table S2, and Figure S1C and Table S3 for A, B, and C, respectively.

Figure 2 shows the evolution of Raman peaks upon the application of external hydrostatic pressures for p-ZnO (Figure 2A), ZMO (Figure 2B), and ZCO (Figure 2C), respectively. It is noted that, for clarity, only representative pressures selected from Figures S1A–S1C are displayed in Figure 2. The results exhibit an apparent trend of frequency blue-shift of all phonon modes with increasing pressure, albeit that subtle differences in details are also evident. For p-ZnO (Figure 2A), the blue-shift of all phonon modes, including *E*_2_^high^-*E*_2_^low^, *A*_1_(TO), *E*_1_(TO), *E*_2_^high^, 2*B*_1_^low^, and *E*_1_(LO) mode proceed monotonically up to 8.3(1) GPa. Moreover, these frequency shifts are accompanied by intensity quenching and line width broadening, which may be attributed to pressure-induced translational crystal asymmetry or bonding anharmonicity. As the pressure being increased to 8.9(1) GPa, the 2*B*_1_^low^ and *E*_1_(LO) phonons vanish completely and are replaced by two phonons appearing at around 545 and 587 cm^−1^, which were identified by Decremps et al.^12^ as the RS(TO) and RS(LO) modes of the RS phase. The appearance of the RS(TO) and RS(LO) modes reflects that the onset pressure of the WZ-to-RS structural transformation occurs at ∼8.9(1) GPa, which is in excellent agreement with the previous high-pressure studies.^40^^,^^41^^,^^42^^,^^43^^,^^44^

Further increase the pressure to above 9.1(1) GPa, the *E*_2_^high^-*E*_2_^low^, *A*_1_(TO), and *E*_1_(TO) phonons disappear completely and the three phonons, namely *E*_2_^high^, RS(TO), and RS(LO), dominate the entire Raman spectrum. The *E*_2_^high^ phonon of the WZ phase disappears at around 10.5(1) GPa, featuring the strong evidence of a pressure-induced metallic phase transition.^43^^,^^44^ As such, the present results evidently indicate that in the pressure range between 8.9(1) and 10.5(1) GPa the WZ and RS are coexisting in the system and the metallic phase transition should be accompanied by the WZ-to-RS phase change as previously described.^11^^,^^12^^,^^20^^,^^45^^,^^46^ Furthermore, the completion pressure of around 10.5(1) GPa for the WZ-to-RS phase transition in p-ZnO is consistent with that obtained by high-pressure XRD measurements reported by Lin et al.^3^

For ZMO (Figure 2B), similar pressure-dependent blue-shift accompanying with continuously intensity quenching and linewidth broadening of all phonon modes are evident up to 8.4(1) GPa. However, unlike that seen in Figure 2A for p-ZnO, although *E*_2_^high^-*E*_2_^low^ and *E*_1_(TO) modes appears to have vanished, the *A*_1_(TO), *E*_2_^high^, DOS, Mn LVM1, and Mn LVM2 modes can still be unambiguously identified even when the pressure is increased to 10.1(1) GPa, indicating that the system remains predominantly WZ-structured. Interestingly, as the pressure is further increased to 11.1(1) GPa, all of the WZ phonon modes suddenly disappeared and the broad band between 500 and 650 cm^−1^ could be neatly deconvoluted into two RS(TO) and RS(LO) modes centering at around 547 and 592 cm^−1^ with about the same intensities. In contrast to that observed in Figure 2A for p-ZnO, there appears to be no pressure range wherein the WZ and RS phases coexist, suggesting that the pressure-induced WZ-to-RS phase transition in ZMO may prevail differently from that in p-ZnO. The phase transition pressure suggested by the Raman results is also substantially different from that obtained by ADXRD, wherein the onset pressure of WZ-to-RS phase transition was significantly lowered to about 7.35 GPa.^3^ This, however, may be understood within the context of the generally conceived nucleation kinetics prevailing in the pressure-driven WZ-to-RS phase transition.^47^ In this scenario, the RS phase firstly nucleates within the bulk of the parent WZ phase and extra energy (*PdV* work) must be put in to stabilize the RS nuclei before the transition can occur. Moreover, since the WZ-to-RS phase transition ZMO has been reported to follow the “hexagonal path”,^3^ as such, the vibrational behaviors between Zn and O atoms exhibiting in Raman spectra preserved the hexagonal structured features of WZ phase until the majority of materials convert to the newly formed RS phase.

For ZCO, as shown in Figure 2C, the pressure-dependent frequency shift for all of the phonon modes behave similarly to that exhibited in p-ZnO up to 7.8(1) GPa. Nevertheless, with the pressure being increased to 8.2(1) GPa, the DOS, Co LVM, and *A*_1_(TO) phonons all diminished, while the *E*_2_^high^ mode remains visible accompanying with the RS(TO) and RS(LO) modes appearing at around 538 and 587 cm^−1^, respectively. As the pressure is further increased to 9.3(1) GPa, the *E*_2_^high^ phonon of the WZ structure vanishes completely and the remaining Raman spectrum can be precisely deconvoluted to the RS(TO) and RS(LO) modes. Thus, for ZCO the onset pressure of WZ-to-RS phase transition can be regarded as being around 8.2(1) GPa and the two phases coexist up to 9.3(1) GPa. Comparing to the results described previously for p-ZnO and ZMO, it is suggestive that, unlike Mn-doping, Co-doping doesn’t alter the general behavior of WZ-to-RS phase transition, although it does reduce the onset-to-complete pressure range from 8.9(1) to 10.5(1) GPa for p-ZnO to 8.2(1) to 9.3(1) GPa. The onset and completion pressures of WZ-to-RS transition are slightly different from those determined by the *in situ* ADXRD method,^3^^,^^48^ presumably due to the fact that Raman mainly probes the lattice dynamics and is more sensitive to the local environments while X-ray measures the average structural parameters. Nevertheless, the observation that ZMO may take different WZ-to-RS transition path is quite consistent with that determined by the ADXRD method,^3^ wherein the pressure-dependencies of *c/a* ratio and internal structural parameter (*u*) for ZMO were found to behave differently from that for p-ZnO and ZCO. The question to be asked is: “does this have anything to do with the observed LVMs?

**Figure 3:**
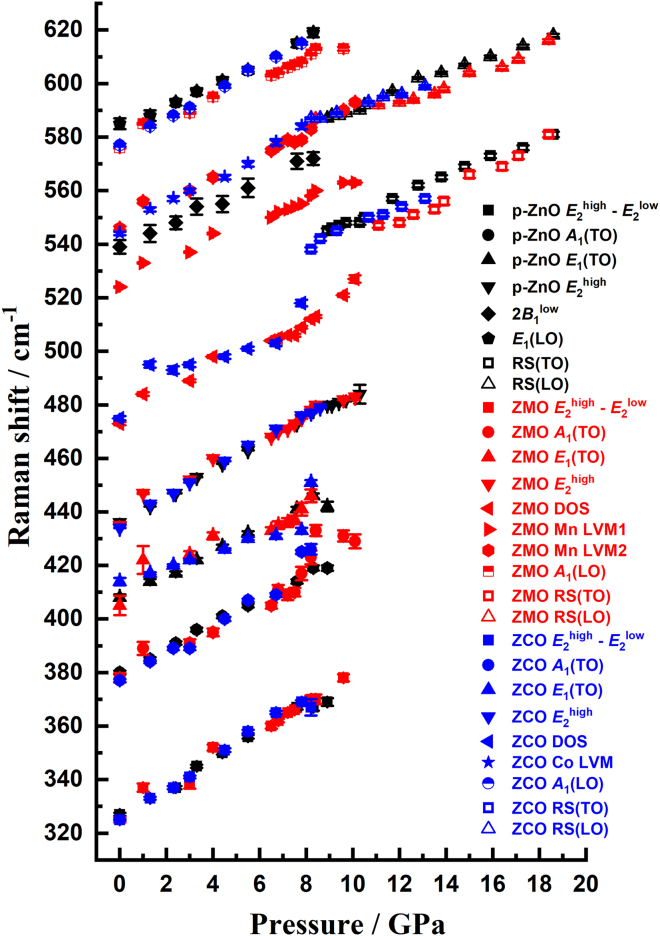
Pressure dependence of Raman modes Pressure dependence of observed optical phonon frequencies. See also Figure S1A and Table S1, Figure S1B and Table S2, and Figure S1C and Table S3 for WZ ZnO, Zn_0.98_Mn_0.02_O, and Zn_0.98_Co_0.02_O, respectively. Data are presented as mean ± standard deviation (SD).

To further explore this issue, Figure 3 shows the pressure dependences of the phonon frequencies for p-ZnO, ZMO, and ZCO, respectively. The detailed numbers are listed in Tables S1–S3. It is evident from Figure 3 that the frequency shifts of all phonon modes exhibit a quasi-linear dependence to the applied pressure, indicating a rather homogeneous compressive deformation in the lattice. The corresponding pressure coefficients (*dω*_*i*_/*dp*) and Grüneisen parameters (*γ*_*i*_) (Equation 2) for each respective phonon modes are also listed in Table 1. According to the simple homogeneous orthorhombic shear strain path proposed for WZ-to-RS transition in A^*N*^B^*8-N*^ semiconductors,^12^^,^^42^^,^^43^ one would expect to see an intermediate phase isomorphic to hexagonal MgO (*h*-MgO) and a negative *γ* reflecting phonon softening of certain mode.^49^ However, neither an intermediate *h*-MgO phase nor a negative *γ* is observed here, indicating that the pressure-induced WZ-to-RS phase in ZnO might not be merely governed by simple homogeneous orthorhombic shear strain. Next, we note that Mn- and Co-doping do have some noticeable effects on *dω*_*i*_/*dp* and *γ* of the phonon modes, in particular the prominent *A*_1_(TO) and *E*_2_^high^ modes.^12^ Namely, comparing with p-ZnO, Mn-doping appears to reduce *dω*_*i*_/*dp* of the *A*_1_(TO) and *E*_2_^high^ modes, while Co-doping has resulted in the opposite. Both Mn- and Co-doping, nevertheless, reduces *γ* of the two phonon modes. Although the former effect of the reduced (increased) *dω*_*i*_/*dp* may explain why Mn-doping (Co-doping) has postponed (facilitated) the WZ-to-RS phase transition to take place at higher (lower) pressure, it cannot reconcile the doping effect on *γ*. Alternatively, the fact that the two pronounced local vibration modes, Mn LVM1 and Mn LVM2, resulted from Mn-doping are having smaller *dω*_*i*_/*dp* and larger *γ* than the Co LVM induced by Co-doping, suggests that Mn LVM1 and Mn LVM2 are more robust to the applied pressure than the Co LVM, which might have played a role in WZ-to-RS phase transition here. Previously, based on the pressure-dependent *c/a* ratio and internal structural parameter *u*(*P*) obtained from ADXRD, it was concluded that the WZ-to-RS phase transition of p-ZnO and ZCO followed the “tetragonal path”, while ZMO took the “hexagonal” path.^3^^,^^48^ Structurally, with increasing pressure, the “tetragonal path” is characterized by first gradually opening the hexagonal angle of the WZ structure from 60° to 90° followed by *u*(*P*) changing from 0.375 to 0.5 when the RS phase appears. For the “hexagonal” path, Zn and O atoms moves closer along the *c*-axis and *u*(*P*) changes from 0.375 with increasing pressure first. Upon reaching *u*(*P*) = 0.5, the hexagonal angle of the WZ structure opens from 60° to 90°, leading to the appearance of RS phase. In view of this scenario, the more robust Mn LVM1 and Mn LVM2 modes might have stabilized the in-plane triangular coordination between the Zn and O atoms until c/a reduces from 1.61 to 1.29 and *u*(*P*) increases from 0.375 to 0.5 to accompany with WZ-to-RS phase transition. The sudden opening of the in-plane hexagonal angle is also consistent with the absence of phase coexistence pressure range in ZMO observed here. Another interesting feature to be noted in Figure 3 is that the Mn LVM2 and Co LVM appear to be following the same pressure dependence even beyond the WZ-to-RS phase transition and turning into the same RS(LO) mode. It is suggestive that the appearance of these dopant-induced LVMs may have been responsible for triggering an earlier onset of the WZ-to-RS phase transition. However, for ZMO, the accompanying appearance of the additional Mn LVM1 could have further altered the path of the WZ-to-RS phase transition.

**Table 1:** Linear pressure coefficients (*dω*_*i*_/*dp*) and mode Grüneisen parameters (*γ*_*i*_) for WZ ZnO, Zn_0.98_Mn_0.02_O, and Zn_0.98_Co_0.02_O, respectively

	phonon mode (*i*)	*dω*_*i*_/*dp* (cm^−1^/GPa)	Grüneisen parameter (*γ*_*i*_)
ZnO	*E*_2_^high^ - *E*_2_^low^	4.94	2.54
(*B*_0_ = 168 GPa)[12]	*A*_1_(TO)	4.52	2.00
	*E*_1_(TO)	4.22	1.74
	*E*_2_^high^	4.80	1.85
	2*B*_1_^low^	4.07	1.27
	*E*_1_(LO)	4.14	1.19
Zn_0.98_Mn_0__.__02_O	*E*_2_^high^ - *E*_2_^low^	5.17	2.50
(*B*_0_ = 157 GPa)[39]	*A*_1_(TO)	4.23	1.76
	*E*_1_(TO)	3.45	1.34
	*E*_2_^high^	4.57	1.65
	DOS	4.11	1.36
	Mn LVM1	3.73	1.12
	Mn LVM2	4.33	1.24
	*A*_1_(LO)	3.83	1.04
Zn_0__.__98_Co_0__.__02_O	*E*_2_^high^ - *E*_2_^low^	5.53	2.02
(*B*_0_ = 119 GPa)[12]	*A*_1_(TO)	5.92	1.87
	*E*_1_(TO)	3.45	0.99
	*E*_2_^high^	5.19	1.42
	DOS	3.99	1.00
	Co LVM	4.85	1.06
	*A*_1_(LO)	4.91	1.01

Previous theoretical calculations by the Hartree-Fock method,^50^ the local-density and generalized gradient approximations (LDA and GGA)^51^ and the first-principles,^52^ in addition to successfully reproducing the experimentally observed onset pressure of the WZ-to-RS phase transition in ZnO, had all indicated that the enthalpy difference between the ambient WZ and high-pressure RS phases was only about 0.15 eV/pair, which is in fact much smaller than the threshold value of 0.25 eV/pair for homogeneous phase transformation.^52^ As mentioned previously,^47^ since the phase transition may not occur as a single homogenous domain, the enthalpy barrier for the WZ-to-RS phase transition in p-ZnO, ZMO, and ZCO reported here is likely dominated by the energy needed to create the nucleation region of the new phase before the rest of a small domain transformed in a cascading fashion. In this perspective, the lattice distortion and associated strain distribution landscape within the material during the pressurizing process might be also playing a pivot role in determining the eventual transition path. Especially, as indicated in Figure S2, the unit cell volume difference between WZ and RS phases at the onset pressure are 16.2, 16.5, and 17.2% for p-ZnO, ZMO, and ZCO, respectively.^3^^,^^48^ If we consider the WZ-to-RS phase transition as an adiabatic process, then the total enthalpy change (Δ^o^*H*) associated with these pressure-driven phase transitions may be estimated as follows:^44^ Δ^o^*H* ≈ *P*_t_ (-Δ*V*), wherein *P*_t_ is the phase transition pressure described previously.^3^^,^^48^ The values of Δ^o^*H* obtained from the results shown in Figure S2 are 24.4, 16.8, and 15.5 kJ mol^−1^ for p-ZnO, ZMO, and ZCO, respectively. These values correspond approximately 0.25, 0.17 eV, and 0.16 eV/unit cell (or 0.13, 0.085, and 0.08 eV/pair) for p-ZnO, ZMO, and ZCO, respectively. It is interesting to note that the value for p-ZnO is, in fact, very close to that of 0.15 eV/pair obtained by the first-principles calculations.^53^ These results further suggest that the enthalpy barrier, though reflect consistently with the onset pressure of phase transition observed in respective systems, may not directly associate with the transition path.

Alternatively, since this energy has been estimated by the huge unit cell volume shrinkage between the WZ and RS phases, it should be accommodated by the strain energy associated with large lattice distortion^54^ and the interfacial energy between the newly nucleated phase and the parent phase. Thus, in order to gain some insight on this scenario, strain analyses on the respective phase based on Scherrer’s equation were carried out as followings. We fit the widths of the first six diffraction peaks from the main phase during compression by the following equation:^53^

(Equation 1) $${FWHM}^{2}\;\cos^{2}\;\theta ={\left(\frac{\lambda }{d}\right)}^{2}+{\eta_{hkl}}^{2}\;\sin^{2}\;\theta$$

**Figure 4:**
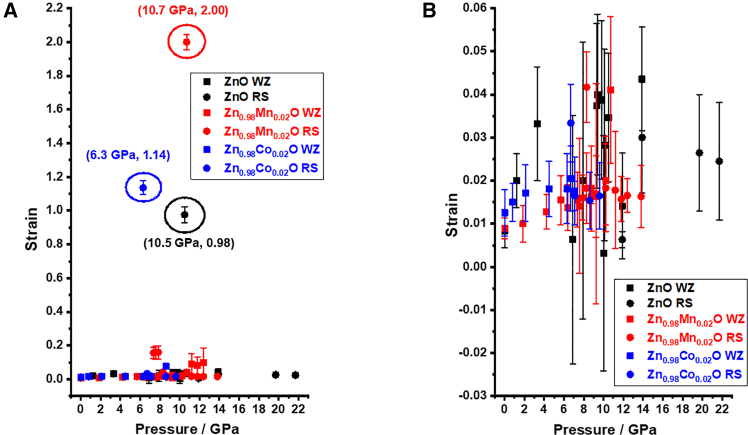
Pressure dependence of strain behavior Strain evolution in the WZ and RS phase during compression. (A) Variations within the range of −0.40–2.20. (B) Only slight variations within the range of 0.01–0.05. Data are presented as mean ± standard deviation (SD).

Wherein *FWHM* stands for the full-width at half-maximum of the diffraction peak on the 2*θ*-scale; *d*, λ, *η*_*hkl*_, and *θ* denote the grain size, X-ray wavelength, lattice distortion or micro-strain, and diffraction angle, respectively. Figure 4A shows the strain in the materials as a function of pressure. It can be seen that before and after phase transition the strain shows only slight variations within the range of 0.01–0.05 (Figure 4B), typical for homogenous deformation. However, as indicated by the circled points in Figure 4A, when the applied pressure reaches *P*_t_ (*P*_t_∼10.5 GPa for p-ZnO and *P*_t_∼6.3 GPa for ZCO) an abrupt jump in strain can be observed, which are 0.98 (Figure S3A) and 1.14 (Figure S3B) for p-ZnO and ZCO, respectively. In contrast, for ZMO, such strain jump does not occur at *P*_t_ (*P*_t_∼7.35 GPa for ZMO), instead it occurs at 10.7 GPa with an enormous strain up to 2.00 (Figure S3C). This is a symbolic phenomenon caused by tetragonal distortion.^2^ During the compression process, the hexagonal angle of the WZ structure, which initially is 60°, opens up to 90°, leading to a sudden increase in strain. Simultaneously, this is accompanied by the appearance of Raman modes coming from the newly formed RS phase,^3^^,^^48^ as seen in Figures 2A and 2C. On the other hand, if the transition is following the hexagonal path, the Zn-O pairs are compressed and shift along the *z* axis. As a consequence, the *u* value increases from initial 0.375 to 1/2, and the *c*/*a* ratio decreases from 1.61 to 1.29 when pressure reaches *P*_t_. In this scenario, each zinc atom (or oxygen atom) remains at the center of a tetrahedron formed by three oxygen atoms (or zinc atoms) at *P*_t_. As the pressure is further increased to beyond *P*_t_, the hexagonal angle of the WZ structure opens from 60° to 90°, expanding the angle at the center of the triangle with the corresponding atoms undergo horizontal movement and the structure transforming to that of the RS phase. Namely, for the hexagonal path phase transition, the feature of sudden increase in strain caused by the 60°–90° opening of the hexagonal angle of the WZ structure typically takes place at pressure higher than *P*_t_. Similar to the tetragonal case, the opening of the hexagonal angle is also accompanied by the appearance of Raman modes associated with the RS phase, which is exactly what was seen in Figure 2B. In addition, within the context of the aforementioned discussion, it would imply that the existence of the Mn LVM1 (Figure 3) might have been the primary reason of suppressing the emergence of the RS(TO) mode, which, in turn, dragged the final step of WZ-to-RS phase transition. That is the opening of the hexagonal angle, which actually involves the in-plane atomic movement and, thus, is more relevant to the TO vibrational mode.

Apparently, the WZ-to-RS phase transition in ZnO is notably sensitive to the introduction of dopants such as Mn, which alter both the structural and electronic properties of the host lattice. In particular, Mn doping can shift the critical transition pressure and modify the transformation pathway. Our results indicate that the partial replacement of Zn^2+^ (3*d*^10^) by Mn^2+^ (high-spin 3*d*^5^) modifies local bonding environments in a manner traceable through Raman scattering, X-ray diffraction, and complementary spectroscopic techniques. The ionic radius of Mn^2+^ in tetrahedral coordination is approximately 0.66–0.67 Å, compared to 0.60 Å for Zn^2+^.^29^ This size discrepancy introduces lattice distortions when Mn is substituted into ZnO, as confirmed by our ADXRD measurements on 2% Mn-doped ZnO.^48^ These measurements reveal slight (∼0.1–0.2%) yet measurable increases in the lattice constants *a* and *c* relative to undoped ZnO, consistent with other reports of transition-metal doping in ZnO.^55^ Such structural perturbations are highly relevant to high-pressure behavior because they influence the crystal’s response to external forces. Moreover, the valence state of Mn is typically Mn^2+^, preserving overall charge neutrality while introducing partially filled 3*d* orbitals into a matrix that otherwise contains fully occupied 3*d* states in Zn^2+^. This contrast in electronic configuration leads to changes in local electron density and bonding interactions, which can in turn reinforce or destabilize specific phonon modes under compression. For instance, the partially occupied *d*-orbitals of Mn are known to enhance electron-phonon coupling locally, potentially shifting the energies of vibrational modes and affecting how pressure is distributed throughout the lattice. The presence of Mn-related impurity levels within the ZnO band gap has also been documented, illustrating how doping can introduce additional electronic states that modify the vibrational and optical properties.^56^ Our high-pressure Raman studies highlight the emergence of a distinct local vibrational mode (LVM1) at ∼524 cm^−1^ associated with Mn doping. This local mode acts as a spectral marker of Mn-O bond distortions, offering insight into how the dopant’s larger ionic radius and modified bonding environment redistribute stress under increasing pressure. Notably, undoped ZnO undergoes the WZ-to-RS transition around 9–10 GPa, though precise values vary according to experimental conditions. In contrast, our measurements reveal that Mn-doped samples can exhibit an onset of this transition shifted by as much as 3–4 GPa, demonstrating a re-engineered pathway attributable to the combined effects of ionic-size mismatch, partial 3*d* occupancy, and the specific local distortions that result from Mn incorporation. These observations collectively suggest that Mn doping stabilizes—or, in some cases, destabilizes—certain configurations of the ZnO lattice, thereby altering the nucleation of the rocksalt phase. The LVM1 at 524 cm^−1^ effectively signifies localized reconfigurations tied to Mn substitution, serving as a real-time probe of how external pressure is accommodated by the doped lattice. Such mechanistic insights underscore the critical role played by impurity size, electronic structure, and local vibrational signatures in defining high-pressure phase transitions. In this way, integrating Raman, X-ray diffraction, and electronic structure analysis affords a coherent picture of how Mn substitution modifies both the local and extended lattice properties of ZnO, ultimately paving a different pathway toward the WZ-to-RS phase transformation.

The influence of Mn doping on the phase-transition pathway in ZnO could be further elaborated by considering both the experimental findings presented here and the context established for broader family of II-VI semiconductors. Previous work on CdTe has shown that substituting Mn into the host lattice significantly lowers the pressure at which the zinc-blende (ZB) structure converts to RS, whereas substituting Zn in the same system elevates the transition pressure.^57^ This behavior has been attributed to the strong hybridization between Mn 3*d* orbitals and the anion *p* states, which weakens the tetrahedral bonding network and renders the structure more susceptible to pressure-induced distortion. In the case of ZnO, it is widely accepted that the degree of *p*-*d* interaction plays a critical role in determining the electronic and structural characteristics of transition-metal-doped samples.^58^ Our experimental observations-particularly the high-pressure ADXRD patterns and Raman spectra-suggest that Mn doping alters the onset pressure for the B4-to-B1 transition and may introduce intermediate structural features that are not evident in undoped ZnO under similar conditions. The relatively strong Mn 3*d*- O 2*p* hybridization, analogous to that seen in other Mn-doped II-VI systems, is likely responsible for these modifications to the transition pathway. Although detailed quantitative analysis is needed to confirm whether these features represent entirely new phases or a shifted version of the known B4-to-B1 mechanism, our findings are consistent with the hypothesis that Mn doping increases the crystal’s susceptibility to compression and induces additional structural distortions. This interpretation is further supported by electronic structure calculations in related systems, which reveal that transition-metal dopants such as Mn can introduce localized 3*d* states that hybridize with the anion *p* orbitals and modify the energy landscape for structural transformations.^59^ By situating our results within this broader theoretical and experimental framework, we highlight the possibility that the phase-transition route of Mn-doped ZnO is distinct in the sense that it may follow a modified pathway-including potential intermediate phases-rather than simply exhibiting a uniform shift in the critical pressure. We will provide additional quantitative evidence from our high-pressure measurements in the final manuscript to strengthen this argument and more clearly illustrate the nuanced role of Mn doping in shaping the high-pressure behavior of ZnO.

### Conclusion

We carried out systematic Raman scattering investigations on pristine ZnO, Zn_0.98_Mn_0.02_O, and Zn_0.98_Co_0.02_O to unveil the effect of transition metal doping on the path of pressure-driven wurtzite-to-rocksalt phase transition. The results indicated that Mn-doping evidently resulted in two impurity-induced local vibration modes with rather pronounced intensity, while Co-doping only induced one modest local vibration mode, presumably due to the ionic size effect. Detailed pressure-dependent analyses on each respectively phonon modes suggested that the existence and robustness of the impurity-associated local vibration modes may have played a prominent role in determining the specific path of the pressure-induced phase transition in ZnO. In particular, from the strain analyses, we observed an abrupt strain burst originated from the opening of the hexagonal angle of the parent wurtzite phase immediately prior to transforming into the rocksalt phase. Finally, the results also suggest that the appearance of the 524 cm^−1^ Mn LVM1 might be responsible for curating a different path for the pressure-driven WZ-to-RS phase transition in Mn-doped ZnO. The exact nature of the dopant-induced local vibrational modes associated with different doping elements, nevertheless, still require further investigations.

### Limitations of the study

Further measurements with different excitonic wavelengths could allow the collection of the PL and absorption spectra of the samples because the Raman spectra were recorded in the actual experimental set-up.

## Resource availability

### Lead contact

Further information and requests for resources and reagents should be directed to and will be fulfilled by the lead contact, Chih-Ming Lin (cm_lin@phys.nthu.edu.tw).

### Materials availability

This study did not generate unique reagents.

### Data and code availability

• All data reported in this paper will be shared by the lead contact upon request.
• This paper does not report original code.
• Any additional information required to reanalyze the data reported in this paper is available from the lead contact upon request.

## Acknowledgments

This work was partially supported by the 10.13039/501100004663Ministry of Science and Technology of Taiwan, grant nos.: MOST 109-2112-M-007-025, 110-2112-M-007-039, 111-2112-M-007-041, 112-2112-M-007-044, and 111-2112-M-A49-040 and 10.13039/100020595National Science and Technology Council of Taiwan, grant no.: NSTC 113-2112-M-A49-034.

## Author contributions

Conceptualization, C.-M.L., S.-R.J., and J.-Y.J.; investigation and resources, Y.-J.T., Y.-S.H., C.-H.H., B.-S.C., and M.-F.T.; validation and data curation, Y.-J.T., Y.-S.H., C.-H.H., B.-S.C., and M.-F.T.; writing-original draft, C.-M.L., S.-R.J., and J.-Y.J.; writing-review and editing and supervision, C.-M.L., S.-R.J., and J.-Y.J.

## Declaration of interests

The authors declare no competing interests.

## STAR★Methods

### Key resources table

**Table:** 

REAGENT or RESOURCE	SOURCE	IDENTIFIER
**Software and algorithms**		

OriginPro 8.1	Origin Lab Corporation	https://www.originlab.com/

**Other**		

high-pressure Raman measurements	TRIAX 550 spectrometers	TRIAX 550 spectrometer, by Horiba - Product details - Pubcompare

### Experimental model and study participant details

Our study does not use experimental models typical in the life sciences.

### Method details

#### Material preparation

Specimens with the nominal composition of Zn_0.98_Mn_0.02_O used in this study were prepared by conventional solid-state reaction method using MnO_2_, LiCO_3_, and ZnO with the purity better than 99.9% as the starting materials. The powders were mixed and ground with acetone in a zirconium oxide ball mill for 12h. The mixed powders were then calcined in air at 1173 K for 5h. To assure the homogeneous reaction of the starting powders, the grounding and calcined processes were repeated for 3 times. In addition, the solid-state reaction method we used as shown in this work is easy handle and only short pre-process period is needed. We used the chemical-grade powders of cobalt oxide Co_3_O_4_ and zinc oxide ZnO with purity of 99.9% as ingredients. Although the prepared period of our samples using the solid-state reaction method only within a day, the method requires high sinter temperature to both ingredient powders completely reacted into Co doped ZnO compound. The post-annealing treatments at a temperatures 1000°C for 5 days were also completed for high uniformity of our products.

#### Sample characterization

The stoichiometry of the Mn(Co)/Zn ratio in the obtained powders was checked using the energy dispersive X-ray spectroscopy attached in the scanning electron microscope (SEM, Hitachi S-4700).

#### The Raman spectra in rocksalt-structured ZnO

Since both transverse optical (TO) and longitudinal optical (LO) phonons at the Γ-point are forbidden by the cubic (Fm-3m) symmetry in perfect RS-ZnO, first-order Raman spectroscopy alone is insufficient to confirm the formation of the RS phase. We therefore provide a systematic analysis of second-order Raman processes, defect-induced scattering, and doping-induced perturbations, all of which can offer alternative avenues for experimentally probing the RS structure.

Previous calculations confirmed that RS-ZnO maintains dynamic stability in the absence of imaginary vibrational frequencies. The computed PDOS reveals distinct acoustic and optical branches that remain silent in first-order Raman scattering when the crystal symmetry is preserved. However, when we introduce dopants into the RS-ZnO lattice, the resulting structural disorder and local distortions can break the selection rules. This partial lifting of symmetry constraints activates modes previously invisible to first-order Raman measurements. Such effects become especially evident when doping with elements of different ionic radii, such as Mn, or with transition metals that introduce localized *d*-states into the phonon spectrum. The simulations show that these local changes shift the phonon density of states, alter phonon lifetimes, and, in some cases, generate additional vibrational features that are observable in Raman spectra.

In parallel, we analyze second-order Raman features, such as overtones and combination modes, which often appear at higher frequencies. These multi-phonon processes provide indirect signatures of the RS phase in ZnO, even if the fundamental first-order modes remain silent. A careful comparison of undoped and doped RS-ZnO reveals that dopant concentration, ionic size, and electronic configuration collectively determine the extent to which disorder-activated phonons appear and the frequency range in which they contribute. When dopant-induced perturbations are pronounced, additional peaks emerge in the Raman spectrum, consistent with the partial breakdown of strict Fm-3m symmetry.

These findings underscore the importance of examining PDOS data and considering doping strategies when using Raman spectroscopy to identify and characterize RS-ZnO. By showing how elemental substitution modifies the vibrational dynamics, our work provides a framework for interpreting experimental Raman signals and clarifies why pristine RS-ZnO remains largely silent in first-order Raman scattering. We anticipate that this extended discussion, supported by the new PDOS results, will aid in distinguishing RS-ZnO from other possible phases and guide further experimental and computational endeavors to optimize ZnO-based materials for mechanical, optoelectronic, and phononic applications [S1-S3].

#### Raman scattering measurements under ambient conditions

Raman scattering measurements were performed with a confocal micro-Raman system (TRIAX 550). The 532-nm line with a power of 0.2 W from Argon ion laser was focused to about 2 ∼ 4 μm in diameter on the sample surface. The back-scattered signal was collected by a microscopic system and recorded with a JOBIN-YVON SPEX SPECTRUM ONE liquid nitrogen-cooled charge-coupled diode (CCD) detector. All spectra were recorded with a microscope objective and 3 accumulations per second with an integration time of 600 seconds. The power delivered on the sample for each ruby fluorescence and Raman spectrum was ∼50 mW. With the diameter of about 2∼4 μm for the focused beam spot on the sample surface, the excitation power density was estimated to be about 2.5 × 10^5^∼1.0 × 10^6^ W/cm^2^. It is noted that the relatively high-power density delivered to the sample surface may induce local heating effect and lead to substantial thermal expansion. Indeed, hence the frequency down-shift of the Raman scattering phonon modes was observed. Wavenumbers were accurate to ± 1 cm^−1^ as determined from plasma emission lines. The Jandel Scientific Peakfit computer program was used in deconvolving the obtained Raman spectra to extract information, such as the band position, band intensity (i.e. band height), band area (i.e. integrated area) and band width (i.e. full width at half maximum, FWHM), of the corresponding Raman peaks. Lorentzian profiles were used throughout the peak fitting, which were carried out until the square of the correlation coefficients, *r*^2^, were greater than 0.995.

#### Raman scattering measurements under pressure in DAC

The investigated samples were ground into the form of fine powders prior to loading into a symmetric diamond anvil cell (DAC) as previously reported.^48^ The pressure-transmitting medium (PTM) used was the methanol-ethanol mixture with a 4:1 (in volume) ratio. The hydrostatic pressure inside the DAC was determined by using the ruby fluorescence pressure gauge technique developed by Mao et al.^57^ The essence of the technique involves recording the R_1_-line of the luminescence spectrum, which is originated from a pure electronic quantum transition within the Cr^+3^ atoms contained in the tiny ruby crystals dispersed within the gasket hole. By reading the peak position of the *R*_1_ and *R*_2_ fluorescence lines from the ruby embedded in the DAC, a resolution of about 0.1-0.2 GPa in the corresponding pressure can be achieved.

#### The Grüneisen parameter (γ_i_) for bond anharmonicity

Anharmonic properties of solids are usually represented in terms of the Grüneisen parameter *γ*, which in essence is reflected in the pressure-dependence on the frequency of the respective vibration mode. Thus, the Grüneisen parameter (*γ*_*i*_) for the *i*^th^ quasi-harmonic mode of frequency *ω*_*i*_ is defined as:^58^

(Equation 2) $$\gamma_{i}=-\left(\frac{d\;\ln\;\omega_{i}}{d\;\ln\;V\;}\right)=\frac{1}{\beta }\frac{\partial \ln\;\omega_{i}}{\partial P}=\left(\frac{B_{0}}{\omega_{i}}\right)\left(\frac{d\omega_{i}}{dP}\right)$$

Where $B_{0}$ is the zero-pressure isothermal bulk modulus. The *β* parameter is the isothermal volume compressibility and *V* is the molar volume in cm^3^·mole^-1^. In the present study, the mode frequencies as function of the applied pressure for p-ZnO, ZMO, and ZCO were extracted from the respective Raman spectra and fitted by the linear function $\omega_{i}=\omega_{0}$ + $\omega^{\prime }$ *P* to reveal how the bonding anharmonicity of a particular vibration mode evolves with increasing pressure, especially before and near the WZ-to-RS phase transition. It is found that p-ZnO, ZMO, and ZCO all exhibit similar anharmonic chemical bonding characteristics with the anharmonicity depending quasi-linearly on the increasing external pressure in the low-pressure regime. However, for ZMO and ZCO, the *E*_1_(TO) and *A*_1_(TO) modes appear to exhibit some anomalous behaviors near the transition pressure, indicating that it might have been affected by doping. Considering that there is an about 17.8% volume reduction in unit cells during the WS-to-RS transition, drastic lattice distortion and, hence, modifications in vibration behaviors are anticipated. We shall come back to this point later.

### Quantification and statistical analysis

No statistical analyses or significance tests were conducted; accordingly, no asterisks appear in any figure legends.
