# Supplementary material for: Effect of dopant-induced local vibration modes on pressure-driven structural phase transition in Mn- and Co-doped ZnO
Source: iScience. 2025 May 2;28(6):112560. doi: 10.1016/j.isci.2025.112560 (PMC12144407; doi:10.1016/j.isci.2025.112560)
Supplement: Document S1. Figures S1–S4 and Tables S1–S3 [file mmc1.pdf]

iScience, Volume *28*

Supplemental information

Effect of dopant-induced local vibration modes on pressure-driven structural phase transition in Mn- and Co-doped ZnO

Chih-Ming Lin, Yi-Jia Tsai, Yi-Sheng Huang, Chia-Hung Hsu, Bo-Shiuan Chen, Ming-Fong Tai, Sheng-Rui Jian, and Jenh-Yih Juang

Data S1/Methods S1: Key Resources Table - List of chemicals, reagents, instruments, and software used in this study.

Figure S1

C. M. Lin *et al.*

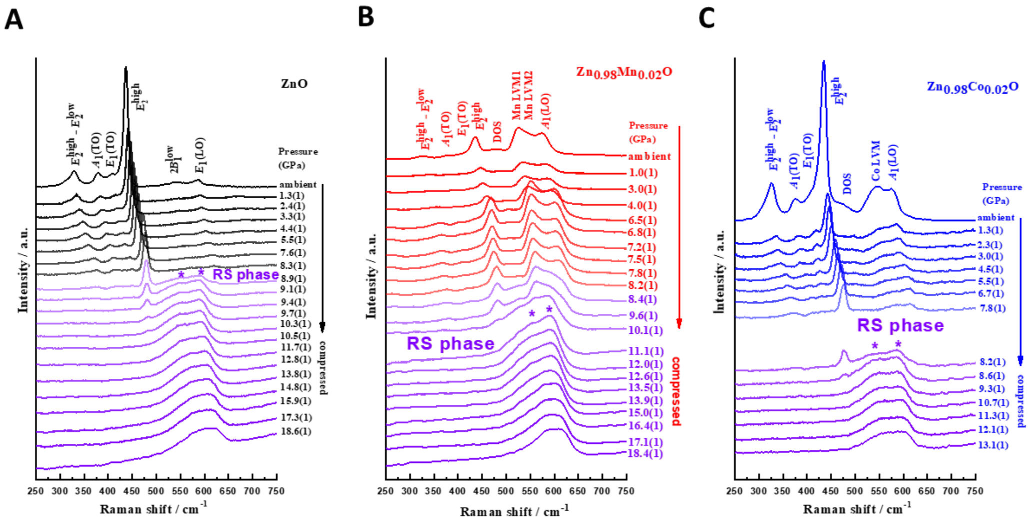

Figure S2

C. M. Lin *et al.*

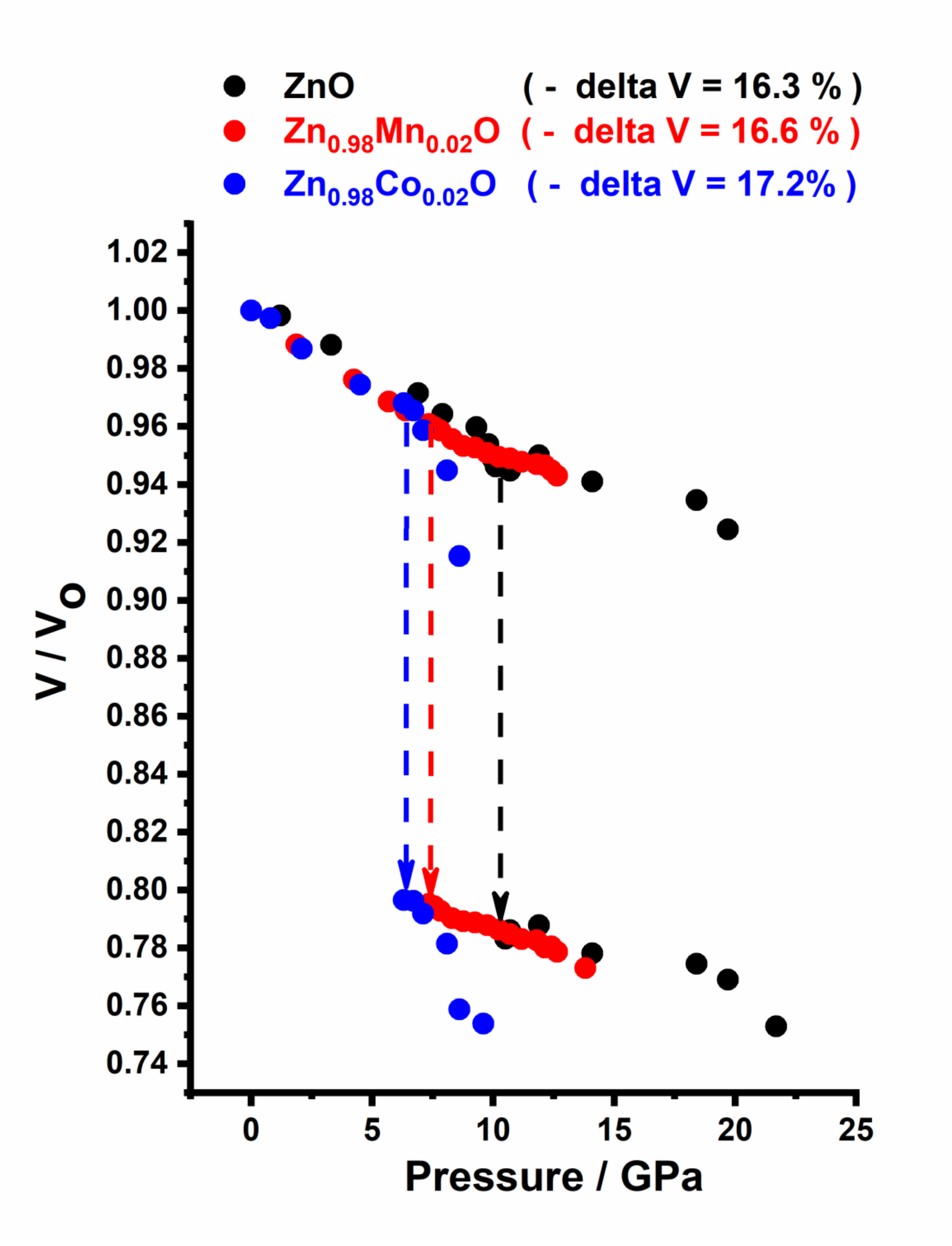

Figure S3

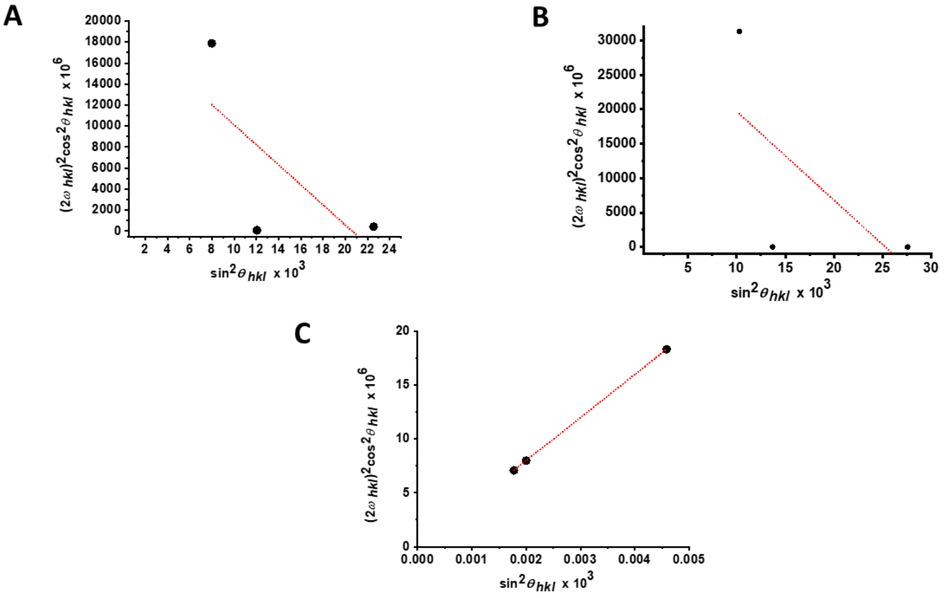
A
20000
18000
16000
14000
12000
10000
8000
6000
4000
2000
0
2 4 6 8 10 12 14 16 18 20 22 24
$(2\omega_{hkl})^2\cos^2\theta_{hkl}$ x 10^6
$\sin^2\theta_{hkl}$ x 10^3
B
30000
25000
20000
15000
10000
5000
0
5 10 15 20 25 30
$(2\omega_{hkl})^2\cos^2\theta_{hkl}$ x 10^6
$\sin^2\theta_{hkl}$ x 10^3
C
20
15
10
5
0
0.000 0.001 0.002 0.003 0.004 0.005
$(2\omega_{hkl})^2\cos^2\theta_{hkl}$ x 10^6
$\sin^2\theta_{hkl}$ x 10^3

Figure S4

C. M. Lin *et al.*

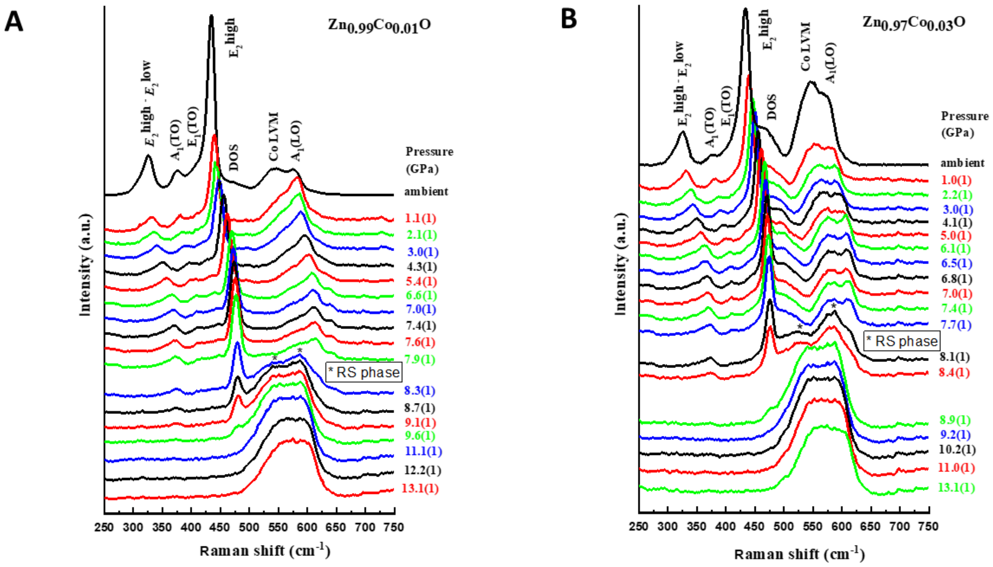

Figure captions

Figure S1. Raman data under pressure

Representative LRS patterns of (A). WZ ZnO, (B). WZ $Zn_{0.98}Mn_{0.02}O$ and (C). WZ $Zn_{0.98}Co_{0.02}O$ at elevated pressures.

Figure S2. Diffraction data under pressure

Pressure dependence of the V/V_0 of ZnO*, $Zn_{0.98}Mn_{0.02}O$**, and $Zn_{0.98}Co_{0.02}O$*, respectively. (*Data for ZnO* and $Zn_{0.98}Co_{0.02}O$*, are taken from Ref.,[3] **Data for $Zn_{0.98}Mn_{0.02}O$** are taken from Ref..[20]). Black, red, and blue filled circles show the strains for ZnO*, $Zn_{0.98}Mn_{0.02}O$**, and $Zn_{0.98}Co_{0.02}O$* at different pressures, respectively. (*Data for ZnO* and $Zn_{0.98}Co_{0.02}O$*, are taken from Ref.,[3] **Data for $Zn_{0.98}Mn_{0.02}O$** are taken from Ref..[20]). Enlarge strain from 0.00 to 0.05 for ZnO*, $Zn_{0.98}Mn_{0.02}O$**, and $Zn_{0.98}Co_{0.02}O$*, respectively. (*Data for ZnO* and $Zn_{0.98}Co_{0.02}O$*, are taken from Ref.,[3] **Data for $Zn_{0.98}Mn_{0.02}O$** are taken from Ref..[20]).

Figure S3. Williamson-Hall (WH) analysis

The black circles show the $(2\omega_{hkl})^2\cos^2\theta_{hkl}$ vs. $\sin^2\theta_{hkl}$ plot for multilayered (A) p-ZnO at 10.5 GPa, (B) ZCO at 6.3 GPa and (C) ZMO at 10.7 GPa

Figure S4. Raman data under pressure

Representative LRS patterns of (A). WZ $Zn_{0.99}Co_{0.01}O$, and (B). WZ $Zn_{0.97}Co_{0.03}O$ at elevated pressures.

Table S1.

ZnO	phonon mode (i) / phonon frequency (cm^{-1})							
Pressure / GPa	E_2^{high} - E_2^{low}	SD(E_2^{high} - E_2^{low})	A_1(TO)	SD(A_1(TO))	E_1(TO)	SD(E_1(TO))	E_2^{high}	SD(E_2^{high})
0.0	327	0.58511	380	0.67757	408	1.16915	436	0.04331
1.3	333	0.54633	385	0.74443	414	0.90898	442	0.03662
2.4	337	0.58448	391	0.67463	417	0.74838	447	0.02977
3.3	345	0.60387	396	0.7597	422	0.80048	453	0.03545
4.4	350	0.67375	401	0.7785	427	0.79236	458	0.03746
5.5	356	0.67753	405	0.67543	432	0.73302	463	0.03769
7.6	367	0.86628	414	0.75297	441	0.90232	473	0.04097
8.3	367	1.23786	419	0.95219	446	0.89316	478	0.0422
8.9	369	1.45608	419	0.99007	442	1.79319	480	0.04721
9.1							480	0.13155
9.4							481	0.25082
9.7							482	0.68302
10.3							484	3.52276
10.5								
11.7								
12.8								
13.8								
14.8								
15.9								
17.3								
18.6								

ZnO	phonon mode (i) / phonon frequency (cm^{-1})							
Pressure / GPa	$2B_1^{low}$	SD($2B_1^{low}$)	E_1(LO)	SD(E_1(LO))	RS(TO)	SD(RS(TO))	RS(LO)	SD(RS(LO))
0.0	539	2.56657	585	1.99358				
1.3	544	3.16416	588	1.95273				
2.4	548	2.38475	593	1.20958				
3.3	554	3.01381	597	1.24829				
4.4	555	3.06469	601	1.38242				
5.5	561	3.44878	605	1.2837				
7.6	571	2.85381	615	1.41747				

8.3	572	2.42273	619	1.73301				
8.9					545	0.95189	587	0.5328
9.1					546	0.73366	588	0.35383
9.4					547	0.72568	588	0.33151
9.7					548	0.68232	589	0.32001
10.3					548	0.70337	590	0.32562
10.5					550	0.65106	592	0.32556
11.7					557	0.7515	597	0.3816
12.8					562	0.76527	602	0.38038
13.8					565	0.77089	604	0.38324
14.8					569	0.84271	607	0.43762
15.9					573	0.88162	610	0.46639
17.3					576	0.90183	614	0.4934
18.6					581	1.02117	618	0.56329

Table S2.

**$Zn_{0.98}Mn_{0.02}O$**	phonon mode (i) / phonon frequency (cm^{-1})									
Pressure / GPa	E_2^{high} - E_2^{low}	SD(E_2^{high} - E_2^{low})	A_1(TO)	SD(A_1(TO))	E_1(TO)	SD(E_1(TO))	E_2^{high}	SD(E_2^{high})	DOS	SD(DOS)
0.0	325	0.9862	378	1.79303	405	3.63357	435	0.14411	473	0.58733
1.0	337	1.58299	389	2.46931	422	5.17256	447	0.27586	484	0.69269
3.0	339	2.37382	391	1.42241	424	1.27741	452	0.19321	489	0.52994
4.0	352	1.10021	395	1.41964	431	1.35436	460	0.14614	498	0.49007
6.5	360	0.98655	405	1.11092	433	1.21775	468	0.11686	504	0.40092
6.8	362	0.64466	411	1.63799	435	1.42945	470	0.10749	505	0.44112
7.2	365	0.67013	409	1.945	436	1.614	471	0.11847	506	0.45563
7.5	366	0.74981	410	1.56623	437	1.59715	473	0.13086	506	0.55672
7.8	369	0.71432	417	2.50723	441	2.38007	475	0.11146	509	0.49056
8.2	370	1.38778	423	2.56684	446	2.44923	478	0.14737	512	0.53453
8.4	370	1.83415	433	2.04408			480	0.16268	513	0.49828
9.6	378	1.32289	431	2.10845			482	0.25414	521	0.6407
10.1			429	2.57205			483	1.1677	525	1.34429
11.1										
12.0										
12.6										
13.5										
13.9										
15.0										
16.4										
17.1										
18.4										

**$Zn_{0.98}Mn_{0.02}O$**	phonon mode (i) / phonon frequency (cm^{-1})									
Pressure / GPa	Mn LVM 1	SD(Mn LVM1)	Mn LVM 2	SD(Mn LVM2)	A_1(LO)	SD(A_1(LO))	RS(TO)	SD(RS(TO))	RS(LO)	SD(RS(LO))
0.0	524	0.2626	546	0.68031	576	0.28405				

1.0	533	0.2806	556	0.66679	585	0.36206				
3.0	537	0.27126	560	0.67213	589	0.31911				
4.0	544	0.28526	565	0.88063	595	0.31977				
6.5	550	0.20776	575	0.57506	603	0.31774				
6.8	552	0.17913	577	0.5947	604	0.28368				
7.2	553	0.16455	579	0.54771	606	0.32344				
7.5	554	0.19847	578	0.6275	607	0.34654				
7.8	555	0.18148	579	0.61581	608	0.29359				
8.2	558	0.2146	583	0.58265	611	0.36414				
8.4	560	0.23447	587	0.61333	613	0.38478				
9.6	563	0.42003	590	0.52387	613	0.67185				
10.1	563	0.73496	593	0.53494						
11.1							547	0.90037	592	0.36764
12.0							548	0.97234	593	0.46205
12.6							551	1.05144	594	0.48279
13.5							553	1.09139	596	0.49407
13.9							556	1.22255	598	0.55677
15.0							566	1.28952	604	0.57067
16.4							569	1.28205	606	0.58529
17.1							573	1.22755	609	0.58504
18.4							581	1.0549	616	0.51406

Table S3.

**$Zn_{0.98}Co_{0.02}O$**	phonon mode (i) / phonon frequency (cm^{-1})									
Pressure / GPa	E_2^{high} - E_2^{low}	SD(E_2^{high} - E_2^{low})	A_1(TO)	SD(A_1(TO))	E_1(TO)	SD(E_1(TO))	E_2^{high}	SD(E_2^{high})	DOS	SD(DOS)
0.0	325	0.52472	377	0.58436	414	0.42222	434	0.05292	475	1.16701
1.3	333	0.62729	384	0.67874	417	0.40142	443	0.05386	495	1.36628
2.3	337	0.54949	389	0.59987	420	0.33705	447	0.04382	493	0.87103
3.0	341	0.67077	389	0.76361	422	0.36396	451	0.04612	495	0.84541
4.5	351	0.62663	400	0.61484	426	0.50351	459	0.04439	498	0.74394
5.5	358	0.58972	407	0.73787	430	0.77104	465	0.04504	501	1.10513
6.7	365	0.6312	409	0.70181	431	1.07002	471	0.05091	503	1.24171
7.8	369	3.11511	425	1.9364	433	0.96348	476	0.09884	518	1.16701
8.2	367	0.52472	426	0.58436	451	0.42222	477	0.08842		
8.6							479	0.05292		
9.3										
10.7										
11.3										
12.1										
13.1										

**$Zn_{0.98}Co_{0.02}O$**	phonon mode (i) / phonon frequency (cm^{-1})							
Pressure / GPa	Co LVM	SD(Co LVM)	A_1(LO)	SD(A_1(LO))	RS(TO)	SD(RS(TO))	RS(LO)	SD(RS(LO))
0.0	544	0.82304	577	0.52071				
1.3	553	0.93355	584	0.60738				
2.3	557	0.78193	588	0.53962				
3.0	560	0.85558	591	0.60371				
4.5	565	1.07519	599	0.61347				
5.5	570	1.21095	605	0.53691				
6.7	578	1.04933	610	0.48266				
7.8	584	0.82304	615	0.52071				
8.2					538	0.80971	587	0.35382
8.6					542	0.79854	587	0.41137
9.3					545	0.63892	589	0.35936

10.7					550	0.73147	593	0.44911
11.3					551	0.68045	595	0.42385
12.1					554	0.77626	596	0.4707
13.1					557	0.80121	599	0.47843

Table caption

“Table S1. [The frequency assignment of WZ ZnO at pressure.], Related to Figure S1A”

“Table S2. [The frequency assignment of WZ $Zn_{0.98}Mn_{0.02}O$ at pressure.], Related to Figure S1B”

“Table S3. [The frequency assignment of WZ $Zn_{0.98}Co_{0.02}O$ at pressure.], Related to Figure S1C”

References

[S1]. Zaoui, A., and Ferhat, M. (2020). Transforming Nonisostructural Wurtzite ZnO and Rocksalt MgO II-VI Compounds into Stable Alloy: The Case of MgZnO. Superlattices and Microstructures, 145, 106623. https://doi.org/10.1016/j.spmi.2020.106623.

[S2]. Alvarado, A., Attapattu, J., Zhang, Y., and Chen, C. F. (2015). Thermoelectric Properties of Rocksalt ZnO from First-Principles Calculations. J. Appl. Phys. *118*, 165101. https://doi.org/10.1063/1.4934522.

Manjón, F. J., Marí, B., Serrano, J., and Romero, A. H. (2005). Silent Raman Modes in Zinc Oxide and Related Nitrides. J. Appl. Phys. *97*, 053516. https://doi.org/10.1063/1.1856222.

[S3]. Sans, J. A., Segura, A., Manjón, F. J., Marí, B., Muñoz, A., and Herrera-Cabrera, M. J. (2005). Optical Properties of Wurtzite and Rock-Salt ZnO under Pressure. MICROELECTRON J *36*, 928-932. https://doi.org/10.1016/j.mejo.2005.05.010.
